# Role of Probiotics in the Management of Patients with Ulcerative Colitis and Pouchitis

**DOI:** 10.3390/microorganisms13010019

**Published:** 2024-12-25

**Authors:** Francesca Bernardi, Fabrizio Fanizzi, Tommaso Lorenzo Parigi, Alessandra Zilli, Mariangela Allocca, Federica Furfaro, Laurent Peyrin-Biroulet, Silvio Danese, Ferdinando D’Amico

**Affiliations:** 1 Gastroenterology and Endoscopy IRCCS, Ospedale San Raffaele, 20132 Milano, Italy; bernardi.francesca@hsr.it (F.B.); fanizzi.fabrizio@hsr.it (F.F.); parigi.tommaso@hsr.it (T.L.P.); zilli.alessandra@hsr.it (A.Z.); allocca.mariangela@hsr.it (M.A.); furfaro.federica@hsr.it (F.F.); danese.silvio@hsr.it (S.D.); 2 Gastroenterology and Endoscopy, Vita Salute San Raffaele University, 20132 Milano, Italy; 3 Department of Gastroenterology, INFINY Institute, INSERM NGERE, CHRU Nancy, F-54500 Vandœuvre-lès-Nancy, France; peyrinbiroulet@gmail.com

**Keywords:** pouchitis, pouch, ulcerative colitis, probiotics, microbiota, IBD

## Abstract

Acute severe ulcerative colitis (ASUC) often requires surgical intervention, such as proctocolectomy with ileal pouch–anal anastomosis (IPAA). While IPAA improves patient outcomes, it can be associated with pouchitis, a common and debilitating complication characterized by inflammation of the pouch. The development of pouchitis is closely linked to dysbiosis—an imbalance in the gut microbiota. Understanding the role of the microbiota in pouch health has spurred interest in probiotics as a therapeutic strategy. Probiotics represent a promising avenue in the management of pouchitis, offering a natural and targeted approach to improving outcomes for UC patients. This review explores the role of probiotics in the management of UC patients, with a specific focus on preventing and treating pouchitis. We compare the microbiota of healthy pouches to those with pouchitis, highlighting key microbial shifts linked to disease onset and discussing the growing evidence for probiotics as a prevention and therapeutic approach. Future directions should prioritize advancing research to optimize probiotic therapies and establish personalized approaches based on individual microbiome profiles, highlighting their significant potential as a promising treatment strategy for pouchitis.

## 1. Introduction

Ulcerative colitis (UC) is a chronic inflammatory bowel disease (IBD) characterized by inflammation of the colonic mucosa. In acute severe ulcerative colitis (ASUC), surgical intervention, such as proctocolectomy with ileal pouch–anal anastomosis (IPAA), is required in up to 20% of cases [1,2]. While IPAA can restore bowel continuity and improve quality of life, it can also be associated with a common complication known as pouchitis [1]. Pouchitis is an inflammatory condition affecting the ileal pouch, and it is associated with symptoms such as abdominal pain, diarrhea, and increased stool frequency [3]. The AGA guidelines classify pouchitis into four distinct categories: intermittent pouchitis, chronic antibiotic-dependent pouchitis, chronic antibiotic-refractory pouchitis, and Crohn’s-like disease of the pouch [3]. The etiology of pouchitis remains unclear, but evidence suggests that an imbalance in the pouch microbiota plays a crucial role [4].

The human gut microbiota is a complex and dynamic community of microorganisms that significantly influences host health. In patients with UC, the composition of the pouch microbiota is often disrupted, leading to dysbiosis—a condition characterized by an imbalance between beneficial and pathogenic microbes [4]. This dysbiosis is thought to contribute to the development and recurrence of pouchitis [4]. Given the central role of the microbiota in pouch health, there is growing interest in the use of probiotics—live microorganisms that confer health benefits to the host—as a therapeutic strategy for managing pouchitis [5].

This review will explore the current understanding of the pouch microbiota in both healthy and pouchitis-affected individuals, highlighting the alterations observed in dysbiosis. Additionally, it will examine the role of probiotics in the prevention and treatment of pouchitis, discussing the evidence supporting their efficacy and the potential mechanisms by which they exert their beneficial effects. The aim is to provide a comprehensive overview of the role of probiotics in the management of patients with ulcerative colitis and IPAA at risk of pouchitis, offering insights into future therapeutic approaches and clinical management.

## 2. Pouchitis

Inflammation in the lining of the pouch (pouchitis) is the most common complication in patients with ulcerative colitis who undergo total proctocolectomy with the creation of a J-pouch and ileo–anal anastomosis [6]. It affects approximately 48% of patients within the first two years following surgery and eventually impacts about 80% of patients over their lifetime [7]. Major risk factors for pouchitis development include the association with extraintestinal manifestations of the disease [8] and, most notably, primary sclerosing cholangitis (PSC) [9].

Pouchitis can be categorized into four types [3]. Intermittent pouchitis is characterized by infrequent episodes with typical symptoms that resolve spontaneously or with antibiotic therapy (ciprofloxacin and metronidazole) [1,3]. Chronic antibiotic-dependent pouchitis generally responds to antibiotic therapy but tends to recur once the treatment course is completed; in this category of patients, the American guidelines recommend the use of probiotic therapy [3]. Chronic antibiotic-refractory pouchitis does not respond to antibiotic therapy and requires biological treatments or corticosteroids [3]. Lastly, Crohn’s-like disease of the pouch should be treated with biological therapy and is defined by specific diagnostic criteria, including the presence of a perianal or other fistula that develops at least 12 months after the final stage of IPAA surgery, stricture of the pouch body or pre-pouch ileum and the presence of pre-pouch ileitis [3,10].

Diagnosis is primarily clinical, marked by an increase in the number of bowel movements (typically, post-operative patients have 4 to 8 daytime bowel movements and 1 or 2 nighttime movements), urgency, and lower quadrant abdominal pain [11]. Biochemical parameters include elevated fecal calprotectin and lactoferrin levels [12,13]. Endoscopic evaluation should be performed when there is suspicion of chronic antibiotic-refractory pouchitis, Crohn’s-like disease, or when atypical symptoms are present [3].

## 3. The Pouch’s Microbiota in Healthy Patients and Pouchitis

The etiology of pouchitis is not fully known [14,15]. However, pouchitis’ pathophysiology is connected to an abnormal immune response toward the commensal microbiota in subjects who are genetically susceptible [4]. Indeed, intestinal microbial dysbiosis is a common feature found in pouchitis [16].

Dysbiosis in pouchitis involves a shift from a balanced microbial environment to one dominated by an imbalance in microbial diversity with a reduction in beneficial bacteria and an increase in harmful microbes, including aerobic and pathogenic species such as *Clostridium perfringens* and hemolytic *Escherichia coli* [17,18,19]. This microbial imbalance leads to an overgrowth of colonic-type flora, altering the biochemical conditions and weakening mucosal defense mechanisms. These changes contribute to the inflammation and development of pouchitis, with dysbiosis playing a key role in driving the immune response and pouch inflammation, explaining why antibiotic treatments are often effective despite the absence of a single identifiable pathogen [17,18,19].

To date, no single etiologic agent has been identified as the cause of pouchitis specifically. However, in a compromised gut or pouch environment, potentially harmful and opportunistic bacteria like Proteobacteria and Bacilli [20], which are normally present at low levels, can proliferate and contribute to inflammation [4,21]. Hence, bacterial families within the expanded phylogenetic gap of pouch patients may have significant roles in pouchitis physiology [22].

Previous studies on normal pouch patients indicated that within the first two years following total proctocolectomy, the fecal microbiota undergoes a time-dependent shift, evolving toward a “colon-like” bacterial community, including nonculturable bacteria, in the ileal pouch [23]. A time-dependent change in the fecal flora of the pouch after total proctocolectomy and stoma closure happens with the increase in the number and diversity of anaerobic and colon-predominant bacteria and the decrease in those of ileum-predominant bacterial species [24]. Using Terminal Restriction Fragment Length Polymorphism (TRFLP, a molecular technique used to analyze and profile microbial communities by detecting variations in the length of DNA fragments generated by restriction enzyme digestion) [25], Falk et al. discovered that the mucosal species in two healthy UC pouches patients were similar to those in the colon [26].

The similarity between the microbiota of a normal pouch and that of a healthy colon remains incompletely understood. The microbiota of a healthy pouch, even after a decade, remains distinct from that of individuals without a history of IBD, indicating it may provide less colonization resistance and fewer nutritional benefits compared to a normal colonic microbiota [4]. Pouch inflammation typically emerges early, with up to 70% of pouchitis cases developing within the first year following restorative proctocolectomy. [27] The rapid symptom relief observed with antibiotic treatment for acute pouchitis supports the theory that bacterial imbalances are central to both the onset and resolution of the condition [28,29,30,31]. However, approximately 30% of patients may experience a progression to chronic pouchitis (CP), with the median time for antibiotic-responsive pouchitis to evolve into CP being less than two years. This highlights the need for ongoing research into microbial dynamics and more effective long-term management strategies [32].

Simultaneously, antibiotic use is associated with worsening dysbiosis, characterized by an increase in potentially harmful bacteria and a reduction in potentially protective taxa. This phenomenon has also been observed in IBD, including reports in pediatric CD patients [33]. The imbalance in the microbiome occurs because broad-spectrum antibiotics reduce the overall bacterial load, leading to inflammation due to immunologic down-regulation. Additionally, repeated antibiotic therapies select for antibiotic-resistant bacteria, altering the microbiota composition and creating a pouch environment that is more susceptible to future episodes of pouchitis [4].

The pouch microbial environment between the inflamed pouch in UC, the healthy pouch in UC, and the healthy pouch in FAP have been proven to be diverse. In a study by Zella et al., TRFLP profiles were consistent across all UC-associated patients (UCP) and differed significantly from those of healthy UC (HUC) and Familial Adenomatous Polyposis (FAP) groups, regardless of the duration or timing of prior antibiotic use. This indicates that the observed dysbiosis may be specific to pouchitis rather than a direct consequence of antibiotic exposure [34]. The exact bacterial populations within the pouch and the dysbiosis associated with pouchitis are not well known. Research using traditional culturing techniques has suggested that pouch effluent contains a higher number of anaerobes compared to ileostomy effluent and that the bacterial communities in the pouch resemble those found in rectal flora [35,36,37]. *Enterobacteriaceae*, *Streptococcaceae*, *Enterococcaceae*, and *Bacteroidaceae* have been found to be the most frequent families of cultivable bacteria adherent to the pouch mucosa, while *Lachnospiraceae*, *Fusobacteiaceae*, *Veillonella*, *Staphylococcaceae*, *Bifidobacteriaceae*, *Eubacteriaceae*, *Bacillaceae*, *Moraxellaceae*, *Burkholderiaceae*, and *Corynebacteriaceae* were the least frequently described ones [38].

A study by Reshef et al. reported a reduced abundance of key taxa in UC pouches compared to FAP pouches. Additionally, the research found that pouchitis was associated with a lower abundance of specific taxa when compared to non-inflamed pouches. *Bacteroides*, *Collinsella*, and 8 genera belonging to the *Lachnospiraceae* and *Ruminococcaceae* families, including *Faecalibacterium*, *Eubacterium*, and *Roseburia* analyzed from fecal and biopsy samples, seem to have a key role in pouchitis [4]. The study correlated disease activity (assessed with clinical, serologic, and fecal markers) with the abundance of specific bacterial taxa and found the most significant correlations using the Pouchitis Disease Activity Index (PDAI) clinical subscore, an index reflecting clinical condition included 11 taxa. *Bacteroides*, *Faecalibacterium*, *Roseburia*, *Coprococcus*, *Eubacterium*, *Clostridium*, *Collinsella*, and *Ruminococcus* negatively correlated with it [4]. Specifically, *Bacteroides* were negatively associated with the PDAI clinical sub-index (R = −0.487 with FDR-corrected *p*-value 0.06), as *Faecalibacterium* (R = −0.345 with FDR-corrected *p*-value < 0.041), *Ruminococcus* (R = −0.334 with FDR-corrected *p*-value < 0.46) and *Roseburia* (R = −0.539 with FDR-corrected *p*-value < 0.002) [4].

Using conventional culture techniques, Nasmyth et al. analyzed fecal flora from 11 pouches and 12 ileostomies, discovering a significant increase in the number of anaerobic bacteria in the samples derived from pouches. In the study pouch, effluent had a greater ratio of anaerobes to aerobes (*p* < 0.05) and greater numbers of *Bacteroides* (*p* < 0.01) and bifidobacteria (*p* < 0.05) compared to ileostomy effluent [39].

A study by Onderdonk et al. examined tissue biopsy samples from patients with and without ileal pouches using electron microscopy and microbiologic culture techniques. The analysis showed that 64 out of 78 samples (82%) grew obligate anaerobic and/or facultative bacteria; meanwhile, 14 samples (17.9%) showed no bacterial growth [40]. Among the positive cultures, 54 contained facultatively anaerobic bacterial species, and 50 yielded obligate anaerobes [40]. The total count of facultatively anaerobic bacteria in samples from pouchitis patients was significantly higher compared to those from the control group. Furthermore, obligate anaerobes were found in a higher proportion of normal pouch samples compared to pouchitis samples, with 4 out of 23 normal pouch samples and 6 out of 12 pouchitis samples (*p* < 0.043) [40].

Komanduri et al. used molecular techniques to compare the pouch microbiota of UC patients with and without pouchitis. By applying length-heterogeneity PCR followed by sequencing, the analysis found that inflamed pouch mucosa exhibited greater species diversity and higher levels of *Fusobacterium varium* compared to healthy UC pouches or normal ileal tissue connected to the colon. In contrast, non-inflamed UC pouches showed a higher presence of *Clostridium*, enteric bacteria, and *Streptococcus* species compared to pouchitis-affected pouches and healthy ileal tissue [41].

The findings of Zella et al. differ significantly from those of Komanduri et al., both in methodology and results. Zella et al. aimed to identify bacterial populations unique to inflamed or healthy UC pouches using molecular techniques, ensuring pouch fecal stasis was constant across all groups [34]. In contrast to Komanduri’s work, which reported an increase in *Fusobacterium* species in inflamed pouches, Zella’s study did not observe this trend [41]. Instead, Zella’s 16S rDNA sequencing revealed different luminal bacterial compositions, with the pouchitis group showing markedly lower levels of *Bacteroidetes* and higher levels of *Clostridia* when compared to the healthy FAP group [34]. This lack of agreement between the two studies highlights the complexity of microbial dynamics in pouch inflammation and underscores the need for further investigation to reconcile these conflicting results. These findings outline how both mucosal and luminal dysbiosis are present in pouchitis, not only in comparison to healthy UC pouches but also to non-IBD pouches, like FAP ones. In line with previous reports, these results lead to the hypothesis that an alteration in the ileal pouch microbiota may be typical to the UC disease state in both the inflamed and non-inflamed UC. Thereby, the ileal pouch microbiota may undergo alterations unique to the UC disease state, regardless of the presence of inflammation [34].

*Bacteroidetes* play a central role in guaranteeing intestine well-being. It has been suggested that a relative decrease in this population might promote the development of inflammation [2,42]. Indeed, the decrease in the phylum *Bacteroidetes* observed in pouchitis is common in UC and CD patients, as proved by multiple molecular surveys of intestinal microbiota in IBD [42,43].

*Bacteroidaceae* and *Clostridiaceae* have been frequently linked to microscopic inflammation of the pouch mucosa, indicating a potential role in the pathogenesis of pouchitis. On the other hand, *Enterococcaceae*, along with possibly *Enterobacteriaceae* and *Streptococcaceae*, may help maintain immunologic balance within the pouch mucosa, where reduced levels could increase the risk of developing pouchitis [2,44].

Although the incidence of *Clostridiaceae* spp. in the pouch mucosa was similar between the CP and normal pouch groups, Scarpa et al. found a significantly higher incidence of *Clostridiaceae* spp. in the chronic/recurrent pouchitis group. Moreover, the presence of *Clostridiaceae* spp. was identified as an independent predictor of chronic/relapsing pouchitis through multiple logistic regression analysis [38].

These results align with those reported by Zella et al. Their DNA sequencing showed that the genus *Clostridium* comprised 9% of the identifiable clones in the UCP pooled sample, whereas it was only 1% in the pooled FAP sample. Additionally, the class *Clostridia* constituted 53% of identifiable clones in the UCP group compared to 21% in the FAP group [34].

Moreover, Zella et al.’s DNA sequencing also demonstrated a significant increase in *Clostridia* in the inflamed pouch, namely among *Clostridium*, *Lachnospiraceae*, and *Roseburia* [34]. The study found bacteria of the genus *Roseburia* in significantly higher numbers in pouchitis compared to FAP pouches and the mucin-degrading genus *Akkermansia* of the phylum *Verrucomicrobia* more prevalent in pouchitis [34]. *Roseburia* are flagellated commensals of the colon with pro-inflammatory gene expression by activating Toll-like receptor 5 (TLR5) induced by flagellin [34]. It is still unknown if *Roseburia* species may increase mucosal inflammation by TLR5 activation [34]. However, high levels of anti-flagellin antibodies, specifically anti-CBir1 antibodies to flagellin of *Clostridium* species, have been associated with pouchitis’ development. A prospective study by Fleshner et al. proved how both pANCA and anti-CBir1 expression may be associated with pouchitis after IPAA. Anti-CBir1 increases the incidence of acute pouchitis (AP) in patients expressing low levels of pANCA and increases the incidence of chronic pouchitis (CP) only in patients with a high level of pANCA expression [45]. Thereby, different forms of pouchitis after IPAA could result from diverse patterns of reactivity to microbial antigens [45,46].

As per the mucing-degrading *Verrucomicrobia* species and *Akkermansia* in particular, a recent large-scale genome-wide association study found that mutations in the MUC19 gene, which encodes mucin proteins, were significantly more frequent in patients with CD [47]. The role of mucin in the protection of intestinal epithelium in IBD has been discussed by many theories that associate mutations, alterations, and degradation of mucins with CD and UC [48,49,50,51]. Thereby, mucin degradation has been hypothesized as a possible factor in pouchitis’ pathogenesis.

When evaluating pouchitis and its relationship to clinical, endoscopic, and histological markers, numerous questions emerge. However, only a few microbial taxa have been correlated with the inflammatory marker CRP (C-reactive protein), suggesting that local changes may occur earlier in the inflammatory process of intestinal inflammation [4]. The absence of a correlation between microbiota composition and the histologic component of the PDAI may be due to the fact that the pouch in a clinically symptomatic patient is not uniformly affected [4]. This novel observation is reinforced by recent studies showing a poor correlation between histologic scores and clinical or endoscopic assessments [4].

An overview of the results of microbiota analysis in pouch and pouchitis is given below (Table 1).

**Figure 1:**
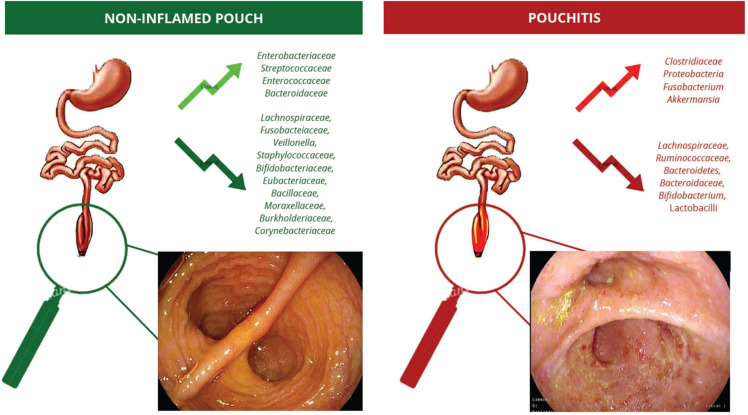
Increase and decrease in bacterial populations in non-inflamed pouch and in pouchitis.

In summary, while the exact cause of pouchitis is still uncertain, growing evidence points to a dysbiosis of the pouch microbiota and an abnormal mucosal immune response as key factors in its pathogenesis (Figure 1).

## 4. Probiotics in Pouchitis

Probiotics are live microorganisms that, when consumed in adequate amounts, confer health benefits beyond basic nutritional content [52]. Probiotics, to be clinically effective, must be resistant to stomach acid and bile, remain metabolically active within the gut microbiota, survive without long-term persistence, exhibit antagonistic properties against pathogenic bacteria, be safe for human consumption, and retain viability after production [53]. The bacteria most frequently linked to probiotic benefic effects include lactobacilli, bifidobacteria, and streptococci [53]. Their benefits include modulation of the gut microbiota, enhancement of the intestinal barrier, and immunomodulation through direct interactions of probiotic bacteria with different immune and epithelial cell types [54].

The American Gastroenterological Association recommends probiotics for the maintenance of remission in chronic recurrent pouchitis, preventing pouchitis in patients who had recurrent episodes of pouchitis previously healed with antibiotics [3]. Contrarily, they do not recommend, and neither are against, the use of probiotics to prevent pouchitis or for patients with rare episodes of pouchitis [3]. The ECCO guidelines align with the first statement, specifying the use of VSL#3 (De Simone formulation) as the recommended probiotic [1]. Furthermore, they advise the use of this formulation in the prevention of pouchitis [1].

### 4.1. Prevention of Primary Pouchitis

Several studies have been conducted on patients with IPAA who had never experienced pouchitis. Yosueda et al. demonstrated the potential role of a probiotic formulation, MIYARI, based on Clostridium butyricum, in the prevention of pouch inflammation. In this study, nine patients received the probiotic at a dosage of 60 mg three times daily, while eight patients received a placebo. After 24 months, only 11% of patients in the probiotic group developed pouchitis, compared to 50% in the placebo group (*p* = 0.07) [55]. Additionally, another probiotic formulation administered one week after ileostomy closure showed efficacy in preventing pouchitis in patients [30]. This formulation, VSL#3 (De Simone formulation), which consisted of a mixture of *lactobacilli* (four strains), *bifidobacteria* (three strains), and *Streptococcus thermophilus*, was administered at a dose of 3 g daily for one year, yielding positive outcomes [30]. Only 10% of patients receiving the probiotic therapy developed pouchitis, compared to 40% in the placebo group (*p* < 0.05) [30]. Moreover, a probiotic containing *Bifidobacterium longum* BB-536 was administered to seven patients, with five patients receiving a placebo [56]. In the probiotic group, only 14% developed pouchitis, whereas 40% of patients in the placebo group did [56]. A larger cohort study by Gosselink et al. demonstrated that the administration of *Lactobacillus rhamnosus GG* (3.0 × 10^11^ live bacteria/day) over three years to 39 patients was effective in preventing pouchitis. In the probiotic group, only 3 out of 39 patients developed pouchitis, whereas in the control group, 27 out of 78 patients developed pouch inflammation (PDAI ≥ 7) [57]. Pronio et al. conducted an open-label randomized controlled trial (RCT) on 12 patients treated with VSL#3 (9.0 × 10^11^ viable lyophilized bacteria/day) for one year vs. 7 patients in the control group [5]. Patients treated with VSL#3 showed a significant reduction in PDAI score after 12 months of follow-up (*p* < 0.01), while controls maintained a stable PDAI score [5]. Moreover, the data from this study indicate that probiotic administration in IPAA patients promotes the expansion of regulatory cells in the pouch mucosa, thus contributing to the establishment of an anti-inflammatory environment [5] (Table 2).

### 4.2. Intermittent/Active Pouchitis

Different probiotic formulations were also employed in studies regarding intermittent or active pouchitis. Gionchetti et al. administered the De Simone formulation to 23 patients with mildly active pouchitis [58]. Of these, 16 patients (69%) achieved remission, while 7 showed no change [58]. This cohort study demonstrated that treatment with De Simone formulation significantly improved clinical, endoscopic, and histologic parameters according to the PDAI, with nearly 70% of patients achieving complete remission [58]. Another cohort study investigated a probiotic formulation consisting of a culture containing live *Lactobacillus* (La-5) and *Bifidobacterium* (Bb-12) [59]. This was administered daily for 4 weeks to 10 patients who had undergone IPAA surgery [59]. The median endoscopic inflammation score was significantly reduced by 50% following the intervention [59]. Additionally, an RCT evaluated the efficacy of probiotics containing *Lactobacillus rhamnosus GG* for the treatment of pouchitis [60]. In this study, only 1 out of 10 treated patients responded to the probiotic, compared to none in the placebo group [60] (Table 2).

### 4.3. Chronic Recurrent Pouchitis

A randomized, placebo-controlled, double-blind trial conducted by Gionchetti et al. demonstrated that patients with chronic recurrent pouchitis who were in clinical and endoscopic remission following one month of antibiotic therapy could significantly benefit from taking the De Simone formulation at a dose of 6 g/day (comprising four strains of *lactobacilli*, three strains of *bifidobacteria*, and *Streptococcus thermophilus*) [31]. Only 3 out of 20 patients receiving probiotics experienced a relapse (15%), compared to all 20 patients in the placebo group who developed pouchitis (100%) (*p* < 0.001) [31]. Furthermore, analysis of stool samples revealed that concentrations of *lactobacilli*, *bifidobacteria*, and *S. thermophilus* increased significantly from baseline levels only in the probiotic group (*p* < 0.01) [31]. Similarly, Mimura et al. evaluated the De Simone formulation at the same dosage (6 g/day) versus placebo in patients with refractory or recurrent pouchitis who achieved remission after a four-week course of ciprofloxacin plus metronidazole, continuing treatment for 12 months or until relapse [61]. In this study, the cumulative remission rate over the 12-month period was 85% in the probiotic group (with only 3 out of 20 patients relapsing) compared to 6% in the placebo group (with 15 out of 16 patients relapsing) (*p* < 0.0001) [61] Table 2.

## 5. Discussion

Despite promising results of probiotic therapy in prevention and intermittent pouchitis, there is still no solid scientific data confirming they can significantly alter the course of the disease, as also reflected in the American guidelines, even though many patients may benefit from it [3].

One important reason for this is that in the conducted studies, different probiotics were tested, each containing a unique combination of bacterial strains, with each strain offering distinct, strain-specific functions [31,55,59,60]. Consequently, there is a lack of scientific evidence to determine which type of probiotic is most effective for administration in a patient who has never experienced pouchitis and is aiming to prevent it versus a patient who has already been affected by pouch inflammation and has successfully undergone antibiotic therapy [4]. This distinction is crucial because antibiotic therapy alters the microbiota, thereby changing the pouch’s microenvironment and potentially requiring a different probiotic approach compared to an antibiotic-naive patient [4]. Therefore, further studies are needed to identify the most suitable bacterial strains to act within the pouch’s microenvironment and prevent inflammation at that site.

At the same time, there is no evidence to determine the optimal duration for effective probiotic therapy. It may be beneficial to conduct studies on the use of probiotics in monthly or annual cycles or on continuous administration over several years. Regarding prevention, the guidelines suggest that in high-risk patients for pouchitis (such as those with primary sclerosing cholangitis associated with ulcerative colitis), lifelong probiotic therapy could be considered, though no studies have yet been conducted on this specific patient population [3].

Nevertheless, probiotics are undoubtedly safe, with their administration not being associated with adverse events; this could serve as a strong incentive to conduct further studies on the subject. However, they remain costly products, and the financial burden would fall on the patient [3].

The De Simone formulation has been the most widely used probiotic in studies found in literature, to the extent that it has been considered within official guidelines [1]. In the two RCTs evaluating the De Simone formulation in chronic recurrent pouchitis, the efficacy of the probiotic in preventing pouch inflammation is considerably evident [31,61]. Bacteria can reduce the inflammation state of the pouch by decreasing levels of pro-inflammatory cytokines (TNF-alpha, IFN-gamma, IL-1alpha) while simultaneously increasing the production of IL-10, an anti-inflammatory cytokine [62]. The other mechanisms, however, remain unknown. We, thus, have evidence that the De Simone probiotic formulation is effective for the pouch microenvironment that has undergone multiple episodes of inflammation and has been influenced by several cycles of antibiotic therapy [31,61].

However, the cost of probiotic therapy remains a significant concern. Therefore, it is essential to expand current knowledge through additional studies to support broader accessibility of probiotic therapy for patients and begin to consider it, in such situations, as a legitimate medication.

Since microbial dysbiosis is a major factor in the pathogenesis of pouchitis, microbiota transplantation has also been explored as a therapeutic approach. However, this has not yielded satisfactory results in any studies conducted so far [63,64]. The reason for this could be that the transplants were performed using healthy colonic microbiota, which may not be appropriate for a pouch, given the known differences in microbial composition between the small and large intestines [65]. Furthermore, colonic metaplasia of the ileal pouch, induced by the new anatomical setting, is associated with chronic and recurrent pouchitis [66]. The next step could be selecting donors of ileal microbiota, such as patients with FAP who have undergone colectomy, patients with an ileostomy, or even an autologous fecal microbiota transplant (FMT) by collecting the transplant material when the ileal pouch is not yet inflamed [67]. No studies have yet been conducted in this direction, potentially due to concerns that the microbiota of these donors may also exhibit dysbiosis given their underlying conditions (FAP, IBD) [67].

The safest option might be a non-donor-derived, synthetic bacterial consortium, possibly administered in pill or suppository form, effectively functioning as a probiotic [67]. Bacterial consortia, defined as complex and specialized mixtures of bacteria, have emerged as highly specific and promising therapeutic agents. These are composed of well-characterized bacterial species selected to function synergistically, mimicking a particular microbial ecosystem [68,69]. Their applications extend to targeted modulation of the intestinal microbiota in complex pathological conditions, even though anti-inflammatory effects are determined by the specific composition of the microbial species [68,69]. Studies have demonstrated their ability to downregulate pro-inflammatory cytokines, including IL-1β, IL-6, IL-8, IL-17, IL-22, TNF-α, and interferon-gamma (IFN-γ), as well as T helper cell subsets such as Th1, Th2, and Th17, which are markedly upregulated in inflammatory states [70]. Furthermore, bacterial consortia have been shown to reduce levels of vascular endothelial growth factor (VEGF), transforming growth factor-beta (TGF-β), and matrix metalloproteinases (MMPs), particularly MMP-2 and MMP-9, thereby contributing to the attenuation of colonic fibrosis [70]. These findings should encourage the initiation of further studies utilizing specific bacterial consortia with anti-inflammatory properties for the treatment of pouchitis.

## 6. Conclusions

The management of patients with UC who undergo IPAA poses significant challenges, particularly due to the frequent occurrence of pouchitis. As our understanding of the pouch microbiota advances, it becomes increasingly clear that dysbiosis plays a pivotal role in the pathogenesis of pouchitis and in exacerbating its chronicity, leading to a substantial impact on patient quality of life.

Probiotics have emerged as a promising therapeutic option in both the prevention and treatment of pouchitis. By modulating the gut microbiota, probiotics can help restore microbial balance, reduce inflammation, and potentially decrease the recurrence of pouchitis. The evidence supporting the efficacy of probiotics, particularly in the form of specific strains and combinations, is growing, although further research is needed to optimize their use and fully understand the mechanisms through which they exert their beneficial effects.

Looking forward, the integration of probiotics into standard care protocols for UC patients with IPAA may offer a targeted and natural approach to managing pouchitis. Additionally, exploring the potential of personalized microbiome-based therapies could lead to more precise and effective interventions.

## Figures and Tables

**Table 1:** Selected evidence on microbiota analysis in pouch and pouchitis.

Authors	Sample Size	Study Design and Endpoints	Main Outcomes
Nasmyth et al. (1989) [39]	29 UC patients (15 with pouch and 14 with ileostomy)	Comparison between ileal ecology after pouch–anal anastomosis or ileostomy	Pouch vs. ileostomy: 8.3 vs. 9 Jog^10^ cfu/mL of total aerobes (*p* < 0.05) Pouch vs. ileostomy: 10.6 vs. 9.6 Jog^10^ cfu/mL of total anaerobes (*p* < 0.05) Pouch vs. ileostomy: 9.8 vs. 5.7 Jog^10^ cfu/mL of *Bacteroides* (*p* < 0.01) Pouch vs. ileostomy: 8.7 vs. 0 Jog^10^ cfu/mL of *Bifidobacteria* (*p* < 0.05)
Onderdonk et al. (1992) [40]	78 patients with pouch or ileostomy	Comparison in microbiologic cultures from normal pouch, pouchitis, indeterminate, normal, and ileostomy patients	OA negative: 4/23 normal pouch vs. 6/12 pouchitis (*p* < 0.043) FA negative: 6/23 normal pouch (*p* < 0.006) vs. 0/12 pouchitis (*p* < 0.05) vs. 5/14 indeterminate (*p* < 0.07) vs. 5/9 normal (*p* < 0.00) vs. 8/20 ileostomy (*p* < 0.03)
Komanduri et al. (2007) [41]	20 UC patients with pouch	Microfloral patterns characterization in pouchitis	*Clostridium paraputrificum* % composition in pouch control: 0.08 *Streptococcus* sp % composition in pouch control: 0.34 *Fusobacterium varium* % composition in pouchitis: 0.12
Zella et al. (2011) [34]	19 patients (9 UCP, 3 HUC, 7 FAP)	Cross-sectional study: comparison of mucosal and luminal flora in UCP, HUC, and FAP pouches using TRFLP and DNA sanger sequencing	*Lactobacillus* and *Streptococcus* TRFLP in FAP vs. UCP at a ratio of 5:1 in mucosa, 3:1 in stool *Clostridium*, *Eubacterium*, and *Roseburia* in HUC vs. UCP at a ratio of 1:15 and in UCP vs. FAP at a ratio of 5:1 *Escherichia*, *Streptococcus*, and various sulfur-oxidizing bacteria in HUC vs. UCP at a ratio of 1:2 UCP vs. FAP: *Firmicutes* (51.2% vs. 21.2%) and *Verrucomicrobia* (20.2% vs. 3.2%), *Bacteroidetes* (17.9% vs. 60.5%) and *Proteobacteria* (9.8% vs. 14.7%)
Reshef et al. (2015) [4]	140 pouch patients (131 UC and 9 FAP)	Prospective study: correlations of microbiota to the PDAI with 16S rRNA gene amplicon pyrosequencing	(*) *Coriobacteriaceae*, *Collinsella* R = −0.611, *p* = 0.001; *Lachnospiraceae*, *Roseburia* R = −0.539, *p* = 0.002 *Bacteroidaceae*, *Bacteroides* R = −0.487, *p* = 0.006 *Ruminococcaceae* R = −0.466, *p* = 0.008 *Lachnospiraceae*, *Eubacterium* R = −0.457, *p* = 0.008; *Lachnospiraceae*, *Coprococcus* R = −0.414, *p* = 0.016; *Lachnospiraceae*, *Clostridium* R = −0.414, *p* = 0.016; *Veillonellaceae*, *Megamonas* R = −0.401, *p* = 0.018; *Lachnospiraceae*, R = −0.367, *p* = 0.033; *Fusobacteriaceae*, *Fusobacterium* R = 0.352, *p* = 0.039; *Ruminococcacea*, *Faecalibacterium* R = −0.345, *p* = 0.041; *Lachnospiraceae*, *Ruminococcus* R = −0.334, *p* = 0.046

UC: Ulcerative Colitis; UCP: UC-associated Pouchitis; HUC: Healthy UC pouches; FAP: Familial Adenomatous Polyposis; PDAI: Pouch Disease Activity Index; OA: obligate anaerobes; FA: facultative anaerobe. TRFLP: Terminal Restriction Fragment Length Polymorphism (*) All *p*-values mentioned in the box are the FDR-corrected *p*-value.

**Table 2:** Use of probiotics in different phases of pouchitis.

Author	Duration	Probiotic	Control	Remission (Probiotic; Placebo)	*p*
***Prevention of pouchitis***					
Yasueda et al. [55]	12 months	*Clostridium butyricum*	Placebo	1/9; 4/8	0.07
Gionchetti et al. [30]	12 months	De Simone formulation	Placebo	2/20; 8/20	<0.05
Brown et al. [56]	6 months	*Bifidobacterium longum* *BB-536*	Placebo	1/7; 2/5	NA
Gosselink et al. [57]	>36 months	*Lactobacillus rhamnosus GG*	Placebo	3/39; 27/78	NA
Pronio et al. [5]	12 months	De Simone formulation	No treatment	12, ↓ PDAI; 7, =PDAI	<0.01
***Intermittent/Active pouchitis***					
Gionchetti et al. [58]	1 month	De Simone formulation	Open-label	16/23	<0.01
Laake et al. [59]	1 month	*Lactobacillus* (La-5) and *Bifidobacterium* (Bb-12)	Open-label	5/10 ↓ endoscopic score	NA
Kuisma et al. [60]	3 months	*Lactobacillus rhamnosus GG*	Placebo	1/10; 0/10	NA
***Chronic recurrent pouchitis***					
Gionchetti et al. [31]	9 months	De Simone formulation	Placebo	17/20; 20/20	<0.01
Mimura et al. [61]	12 months	De Simone formulation	Placebo	17/20; 15/16	<0.0001

↓: reduction; PDAI: Pouchitis Disease Activity Index.

## Data Availability

No new data were created or analyzed in this study. Data sharing is not applicable to this article.

## Abbreviations

UC, ulcerative colitis; IBD, inflammatory bowel disease; ASUC, acute severe ulcerative colitis; IPAA, ileal pouch–anal anastomosis; TRFLP, Terminal Restriction Fragment Length Polymorphism; CP, chronic pouchitis; CD, Crohn’s disease; UCP, UC-associated patients; UCH, healthy UC; FAP, Familial Adenomatous Polyposis; PDAI, Pouchitis Disease Activity Index; AP, acute pouchitis; CP, chronic pouchitis; RCT, randomized controlled trial
